# Sentence Comprehension in Primary Progressive Aphasia: A Study of the Application of the Brazilian Version of the Test for the Reception of Grammar (TROG2-Br)

**DOI:** 10.3389/fneur.2022.815227

**Published:** 2022-05-16

**Authors:** Maria Teresa Carthery-Goulart, Rosimeire de Oliveira, Isabel Junqueira de Almeida, Aline Campanha, Dayse da Silva Souza, Yossi Zana, Paulo Caramelli, Thais Helena Machado

**Affiliations:** ^1^Federal University of ABC (UFABC), Mathematics, Computing and Cognition Center (CMCC), São Bernardo do Campo, Brazil; ^2^INCT-ECCE (Instituto Nacional de Ciência e Tecnologia sobre Comportamento, Cognição e Ensino), São Carlos, Brazil; ^3^Cognitive and Behavioral Neurology Research Group of the Department of Neurology of the University of São Paulo (USP), School of Medicine, São Paulo, Brazil; ^4^Cognitive and Behavioral Neurology Research Group, School of Medicine, Federal University of Minas Gerais (UFMG), Belo Horizonte, Brazil

**Keywords:** TROG, language comprehension, primary progressive aphasia, syntax, sentence comprehension, grammar, morphosyntactic

## Abstract

Sentence-comprehension deficits have been described in patients with primary progressive aphasia (PPA). However, most instruments to address this domain in more detail and in a clinical context have not been adapted and translated into several languages, posing limitations to clinical practice and cross-language research.

**Objectives:**

The study aimed to (1) test the applicability of the Brazilian version of the Test for Reception of Grammar (TROG2-Br) to detect morphosyntactic deficits in patients with PPA; (2) investigate the association between performance in the test and sociodemographic and clinical variables (age, years of formal education, and disease duration); (3) characterize the performance of individuals presenting with the three more common variants of PPA (non-fluent, semantic, and logopenic) and mixed PPA (PPA-Mx) and analyze whether TROG-2 may assist in the distinction of these clinical profiles.

**Methods:**

A total of 74 cognitively healthy participants and 34 individuals diagnosed with PPA were assessed with TROG2-Br. Overall scores (correct items, passed blocks), types, and categories of errors were analyzed.

**Results:**

In controls, block scores were significantly correlated with years of formal education (Spearman's *r* = 0.33, *p* = 004) but not with age. In PPA, age, education, and disease duration were not significantly associated with performance in the test. Controls presented a significantly higher performance on TROG2-Br compared to PPA individuals and their errors pattern pointed to mild general cognitive processing difficulties (attention, working memory). PPA error types pointed to processing and morphosyntactic deficits in nonfluent or agrammatic PPA, (PPA-NF/A), logopenic PPA (PPA-L), and PPA-Mx. The semantic PPA (PPA-S) subgroup was qualitatively more similar to controls (processing difficulties and lower percentage of morphosyntactic errors). TROG2-Br presented good internal consistency and concurrent validity.

**Discussion:**

Our results corroborate findings with TROG-2 in other populations. The performance of typical older adults with heterogeneous levels of education is discussed along with recommendations for clinical use of the test and future directions of research.

## Introduction

Sentence comprehension is a complex language function that goes beyond the identification of single words and their meanings. Additional stages include accessing the argument structure of the verb (transitivity) and its associated thematic roles (who did what to whom); a mapping stage in which thematic roles are assigned to the syntactic positions and the activation of the meaning of the sentence (1, 2). Working memory also plays an important role in sentence comprehension, as the meaning of the sentence and its structure must be held online to be integrated into upcoming information or while a particular mental process or physical action is undertaken (3–5). Sentence length and syntactic complexity are known to modulate the allocation of processing resources for comprehension. Concerning syntactic complexity, noncanonical sentences, such as passives (e.g., *the boy is being chased by the dog*), where the order of the elements is different from subject-verb-object (actives), are thought to demand more from working memory resources. The same can be said about the subordinate sentences, where there is one clause embedded within another (e.g., *The man who is eating is watching the cat*) (3).

Primary progressive aphasia (PPA) refers to a group of clinical syndromes, caused by a neurodegenerative disease. The predominant symptom of PPA is a slow progressive disorder of language abilities, in the absence of significant cognitive, motor, or behavioral impairments (6, 7). There are three recognized PPA subtypes: the semantic (PPA-S), the logopenic (PPA-L), and the non-fluent or agrammatic (PPA-NF/A) variants. In PPA-S, lexical and semantic knowledge are the most impaired features, while the PPA-L is characterized by phonological working memory impairment and word-finding difficulties. In PPA-NF/A, patients have motor speech deficits and/or progressive agrammatism (6, 8–10). Some individuals with PPA do not fit into these three main variants and are usually reported as clinically unclassified or mixed PPA (PPA-Mx) cases (10–12).

Sentence comprehension deficits have been described in patients with PPA. The current consensus criteria (6) recommends the assessment of this function for PPA subclassification and suggests types of tasks, namely, answering “yes”/“no” questions, following directions, or matching oral presented sentences to pictures. However, most instruments used to address this domain in more detail, and in a clinical context, have not been adapted and translated into several languages, posing limitations to clinical practice and for research and cross-language comparisons. A comprehensive morphosyntactic assessment is invaluable to monitor symptom progression in PPA and to devise tailor-made interventions to remediate, reorganize, and/or compensate for grammatical and syntactical deterioration in PPA. Additionally, a thorough assessment of receptive language may support orientations to family and carers and indirectly assist patients in the achievement of communication goals.

Morphosyntactic deficits in PPA-NF/A are more often investigated in production tasks involving connected speech (13–17) (refer to Thompson and Mack (18); Boschi et al., (19), for a review). Nevertheless, many studies reported deficits in the comprehension of grammatically complex sentences (8, 13, 20–24), particularly noncanonical sentences or those containing subordinate and center-embedded clauses. In addition, cleft sentences, such as “It is the man that the women poked” were reported to be differentially impaired in PPA-NF/A compared to other PPA subgroups (23). In PPA-S, although syntax and grammar are generally spared (6, 9, 25), it is not uncommon to find a higher error rate compared to controls in sentence comprehension tasks (8, 11, 26, 27), or even patients performing at the same level as PPA-NF/A (11). Impairments in sentence comprehension in PPA-S are explained in terms of difficulties at the word level, which affect the semantic processing of the lexical components of the sentence (8, 9) and/or, at the sentence level due to the inability to manipulate and combine semantic representations to understand the global meaning of the sentence (26). Although the anterior temporal lobe has not been particularly related to sentence comprehension, some individuals with PPA-S may have atrophy extending to the left posterior temporal regions and/or anterior insula bilaterally, regions involved in syntactic processing in controls (26). Sentence comprehension may also be affected by the progression of neurodegeneration in PPA-S (21). In individuals with PPA-L, difficulties in sentence comprehension are also frequent (6, 25, 28) and their performance maybe even worse than PPA-NF/A (3, 25, 28). In this variant, deficits are usually explained by phonological working memory impairments, related to sentence length, and frequency rather than syntactic complexity (6, 13, 24, 25). Compared to other variants, PPA-L is also more impaired in other domains in the neuropsychological assessment (29, 30) and those deficits (particularly in attention and executive function measures) may impact the performance in language tests.

Different types of tests have been used to evaluate oral sentence comprehension and this ability is usually included in aphasia assessment batteries, such as Boston Diagnostic Aphasia Examination (BDAE) (31), Western Aphasia Battery revised (WAB-R) (32), and Montreal-Toulouse Language Assessment Battery (33). However, a more comprehensive assessment may be necessary to design and evaluate the effects of interventions as well as for monitoring the progression of language symptoms in adults with aphasia and PPA (34, 35). The Token Test (36) is often employed and has been translated and adapted into several languages, including Brazilian Portuguese (37, 38). It evaluates comprehension at the sentence level by asking the participants to execute commands. By manipulating sentence length and, to a lower degree, syntactic complexity, this test provides important information about the influence of phonological short-term memory vs. syntactic deficits on sentence processing. Despite its wide use, the Token Test does not present varied syntactic structures to characterize morphosyntactic deficits, as most sentences are presented in the canonical order. Syntactic complexity is added using lexical terms, such as “except for” or “before doing X”, instead of testing grammatical contrasts, such as reversibility, pronoun reference, and inflections. Besides, it requires active manipulation of tokens which is a disadvantage when testing individuals with ideational apraxia or associated motor disorders which are not uncommon in the progression of PPA. Noncanonical sentences are relevant for the assessment of grammar and syntactic processing in aphasia as well as for the investigations of the neural basis of language comprehension (refer to Walenski et al. (39) for a review and meta-analysis). Additionally, these types of sentences may differentiate PPA-L and PPA-NF/A profiles (3, 22).

The Test for Reception of Grammar (TROG-2) was proposed by Bishop (40) as a comprehensive evaluation of auditory sentence comprehension; this has been widely used in children and adults to characterize and diagnose morphosyntactic deficits. It includes 20 different sentence types (blocks) of four items each, devised to evaluate specific grammar structures and syntactic movements (i.e., reversibility and embedded sentences). Each item is formed by a phrase or a sentence that is read aloud to the participant, whose task is to choose among four pictures the one that best represents the content of the sentence. The foils include a modified lexical or grammatical element in relation to the content of the sentence. The participant is instructed to give his/her answer by either pointing to the chosen figure or saying its corresponding number. The first version of TROG was published in 1983 and slightly modified in 1989 to investigate developmental language disorders. TROG-2 was standardized in a sample of 792 children aged 4 to 16 years and 70 adults from 10 regions across the United Kingdom and consists of a revised version of TROG developed to expand the assessment of syntactic comprehension to samples of older children, secondary students, and adults (TROG-2, pearson clinical.co.uk).

The Test for Reception of Grammar has been utilized in some studies on individuals presenting with PPA. Burrell et al. (41) used TROG to compare patients with PPA-NF/A to patients with progressive supranuclear palsy. Both groups were impaired in this test. Another study demonstrated that patients with motor neuron disease and patients with PPA-NF/A had similar impaired performance on TROG (41). In the study by Knibb et al., patients with PPA-NF/A were impaired in sentence comprehension using TROG. A longitudinal study utilized TROG to monitor sentence comprehension in a patient with PPA-S (42, 43). The authors showed that syntactic abilities remained intact while semantic knowledge suffered degradation over time. The TROG has also been used to monitor therapeutic improvements (44, 45).

Primary progressive aphasia is a rare syndrome, and cross-cultural studies are needed to address the impact of language on its clinical manifestations. There are few studies that investigate the reception of grammar and syntactic processing in the three variants of PPA, and most of these studies were conducted in English-speaking samples. One of the necessary steps to reduce this gap involves the translation and cultural adaptation of tools to evaluate language abilities in different languages. A more comprehensive assessment of grammar contrasts is needed, particularly in languages with rich morphology, such as Portuguese. The great heterogeneity of schooling among older adults also demands a characterization of typical performance to obtain parameters for an accurate diagnosis of language deficits.

In this study, we introduce TROG2-Br, a tool for research and clinical assessment of auditory sentence comprehension for Brazilian Portuguese speakers. Our objectives are as follows: (1) To test the applicability of this tool to detect morphosyntactic deficits in patients with PPA; (2) To investigate the association between performance in the test and age and years of formal education in controls and PPA and disease duration, defined as years from the onset of symptoms, in PPA; (3) To characterize the performance of individuals presenting with the three more common variants of PPA (nonfluent, semantic, and logopenic) and PPA-Mx and analyze whether TROG-2 may assist in the distinction of these clinical profiles.

As TROG2-Br is being used for the first time in research with a large sample of patients and controls with PPA, we also report evidence on the validity of the instrument, namely its internal consistency (the correlation among TROG2-Br blocks as an indication that they are measuring the same psychological construct) and its concurrent validity (correlation between TROG2-Br and the Brazilian version of the Token Test, applied at the same session in controls and PPA). In addition, we suggest a shorter version with five blocks to be investigated and validated in future studies on PPA.

## Materials and Methods

### Subjects

The sample comprised 74 cognitively healthy participants and 34 individuals diagnosed with PPA.

The cognitively healthy controls were participants aged 60 or over, who had completed at least 2 years of formal education, selected from a larger sample that includes teenagers and younger adults with a view to validating and obtaining normative data for the use of TROG-2 in the Brazilian Portuguese speakers [preliminary data collected from Pereira et al. (46) and Oliveira et al. (47)]. They were native Brazilian Portuguese speakers, functionally preserved, with no cognitive-related self-reported deficits. They were recruited from institutions that provide courses and leisure activities to seniors in the greater São Paulo region. Advertisements and information about the study were disseminated in these locations, and participants filled out forms with contact information and were invited by the research team to take part in the study. The study was approved by the Ethics Committee of the University of the City of São Paulo (CAAE 0110.0.186.000-11/Research Protocol 13622453) and all participants signed an informed consent form after receiving full information about the study procedures.

Inclusion criteria for controls were defined based on the guidelines of Mayo Older American Normative Studies (MOANS) (48), for individuals without neuropsychological impairment: (1) absence of active psychiatric or neurological diseases; absence of complaints of cognitive difficulties at the anamnesis, and absence of evidence of disorders that could potentially affect cognition; (2) absence of psychotropic medication in doses that may compromise cognitive functions or suggest a neuropsychiatric disorder; (3) independent living style (no functional incapacity); (4) participants with chronic medical diseases, such as diabetes mellitus or hypertension were included only when receiving regular treatment for these conditions, as attested by their physicians. Exclusion criteria: Cognitive impairment screened with the mini-mental state examination (MMSE) (49) and applying the following education-adjusted scores (50): <20, <24, and <27 points to 1–3, 4–7, and 8 years or more of schooling, respectively; (2) subjective cognitive decline (scores higher than 3 or 5 points in the informant questionnaire on cognitive decline in the elderly (IQCODE) (51, 52); and a score of 6 or more points in the geriatric depression scale (GDS) (53), which is suggestive of depression.

Patients with PPA were recruited from the Behavioral and Cognitive Neurology Outpatient Clinic of Hospital das Clínicas (HC), Federal University of Minas Gerais (UFMG), in Belo Horizonte, Brazil. They were invited to participate in the study after receiving a clinical diagnosis of PPA, by a senior neurologist (PC) in an interdisciplinary consensus meeting. For the diagnosis, clinical history, laboratory, and neuroimaging results, neurological assessment including brief or semi-structured cognitive screening, and speech and language assessment were analyzed against current criteria (6). Patients that met PPA criteria but could not be classified into the three variants were defined as PPA-Mx. Recruitment took place from 2014 to2020. Exclusion criteria were as follows: the first language not being Portuguese; illiteracy or <2 years of formal education; severe sensory and/or motor deficits and severe aphasia, precluding testing with TROG2-Br. The study was approved by the Ethics Committee of the School of Medicine of Federal University of Minas Gerais (CAAE 60390116.9.0000.5149/ Research Protocol 2.018.855) and individuals with PPA and/or legally responsible signed an informed consent form. Speech and language assessment for diagnosis of PPA included a semi-structured interview to evaluate language and motor speech deficits, as well as functionality for communication. Language assessment also included the Boston Naming Test and the following subtests of the BDAE (31, 54): auditory comprehension (commands and complex ideational material), repetition of words and sentences of low and high frequency, automatic speech, reading comprehension of sentences and paragraphs, and narrative writing. The Cambridge Semantic Memory Research Battery (CSMRB) (55–57) and the Reading and Writing tasks of the HFSP protocol (56) were used for the assessment of semantic memory and reading and writing deficits.

## Materials

### The Brazilian Version of the Test for Reception of Grammar-2 (TROG2-Br)

The first author of this paper obtained written authorization from the Pearson Assessment (UK) to translate and culturally adapt the test to Brazilian Portuguese as part of a study investigating language comprehension in frontotemporal neurodegenerative syndromes [CAPES grant BEX 4335/074 (58–60)]. The English version of TROG-2 was translated to Brazilian Portuguese and back-translated to English. Two independent translations followed by two independent back-translation were undertaken. The back-translations were analyzed for compatibility with the original test and inconsistencies were discussed and consensually solved. The final version was analyzed by a committee of experts, including speech and language therapists and linguists, and modifications were proposed to achieve: (1) the correspondence and relevance of syntactic structures evaluated in English and in Portuguese (content validity) and (2) the maintenance of test properties (number of blocks, number of stimuli, and sentence length) to allow for cross-cultural comparison studies. The resulting version was then applied to adult individuals of different levels of education for cultural adaptation and evaluation of test procedures. While being tested with TROG2-Br, these participants commented on each item, providing additional information regarding the suitability of graphic material, and sentences that sounded ambiguous according to target and foils. After this phase, the committee of experts proceeded with minor final adjustments to create TROG2-Br (46). The final version was considered suitable both in terms of language (translation), test administration, content (syntactic structures), and graphic material and was also applied preliminarily in patients with frontotemporal dementia (61) and in neurotypical elderly individuals (47). TROG2-Br sentence stimuli are available upon request to the correspondent author. The stimulus book, manual, and record forms are available from pearson https://www.pearsonclinical.co.uk/.

Twenty syntactic constructions are assessed by TROG-2. According to the manual, the test should be discontinued when a participant fails five consecutive blocks (20 items). However, given TROG2-Br was being applied for the first time in elderly individuals and Brazilian patients with PPA, the 20 blocks (80 stimuli) were assessed for the entire sample. The test score is the number of blocks whose four items were answered correctly (passed blocks). The number of errors per block can be interpreted as indicative of the level of impairment concerning the syntactic structure evaluated in that block: four items (systematic errors) indicate an inability to interpret the sentence construction and reveal severe receptive impairment; two to three items (random errors) indicate difficulty with grammatical constructions and chance performance level; one error (sporadic error) suggests processing difficulties (i.e., limited attention and working memory) but no genuine syntactic deficit in the auditory comprehension of the constructions. The maximum possible overall score is 20 blocks or 80 items. The test was applied according to the manual instructions except for NOT interrupting the test after 5 failed blocks.

As TROG2-Br is a translation and adaptation of TROG-2, it is important to have estimators of its validity and reliability to establish the capacity of the test to measure the underlying construct (grammar comprehension) in the most accurate and consistent way, without much variation by random error. We investigated two aspects: test homogeneity (internal consistency/construct validity) and the equivalence of TROG2-Br to another valid measure of the same construct (concurrent validity). To assess the internal consistency and construct validity, we calculated Cronbach's alpha for the version of 20 blocks. Correct (passed) blocks were coded as 1 and incorrect (failed) ones, as 0. As the original test, the sum of scored blocks was used to quantify the general ability of sentence comprehension of the individual being tested. Cronbach's alpha is one of the ways to quantify the internal consistency of a test, which is an indicator of its construct validity. If the items measure a single psychological construct, the responses must correlate strongly but not perfectly; otherwise, the test loses power in discriminating between individuals performing at the higher or lower level. To estimate the concurrent validity, we applied the Brazilian version of Token Test (36, 37) in a subsample of PPA and controls and evaluated its correlation with TROG2-Br. Token and TROG2-Br were applied at the same session in controls and patients with PPA.

### A Shorter Version of TROG2-Br

Another applicability of Cronbach's alpha is to use it as a parameter to create a shorter version of a test, which is useful to test populations with limited sustained attention or in contexts of time constraints. For this purpose, the items that contribute negatively to the internal consistency are excluded in successive iterations, one at a time. Items with lower or negative contributions to the test are excluded first. For each iteration, a test of internal consistency without that item is determined until reaching a composition of items with maximal internal consistency. Iterations can be done until Cronbach's alpha values remain high or until reaching a predetermined number of items. We followed this procedure using LTM, a package of R (62), to obtain a new estimate of the internal consistency of the shorter version. For the selection of items, we used the data from 21 PPA and 73 controls. We excluded subjects with scores lower than 5 blocks (1 control, 6 PPA-Mx, 3 PPA-S, 2 PPA-L, and 2 PPA-NF/A) in order not to bias the selection of items with extreme results.

### Assessment Procedures

Controls were tested individually at the Human Cognition Lab at UFABC or on the premises of institutions where they were recruited for the study. A typical interview and assessment session lasted for 1 h 30 min and included the following: (1) questionnaires and brief cognitive tests to check if the participant complied with inclusion and exclusion criteria including Addenbrooke's Cognitive Examination Revised (ACE-R) (63–65) assessment with TROG2-Br. A subsample was also tested with the Brazilian version of the Token Test (37), which has been validated for use in elderly individuals in Brazil.

Patients with PPA were assessed in the Behavioral and Cognitive Neurology Outpatient Clinic of HC-UFMG where they were assessed with TROG-2 and a subsample also with the Token Test.

### Statistical Analysis

All computations were performed using SPSS software, version 17 (SPSS INC) (66) and R packages (67).

### Descriptive Statistics

We reported sociodemographic and clinical data on PPA and the control group (CG). As most variables were not normally distributed, we employed nonparametric tests. For between-group comparisons, we employed the Mann–Whitney U test (for 2 independent samples and for *post-hoc* tests) and the Kruskal-Wallis H test (three or more independent samples). For within group comparisons, we used the Wilcoxon *Z*-test. Pearson's chi-squared test was employed to investigate differences between the expected and observed frequencies in categorical variables. Performance in TROG2-Br was reported in terms of correct blocks (blocks in which all four sentences were correctly responded, maximum 20) and of the total number of correct items (maximum 80). We have also analyzed the types of errors that were classified as follows: sporadic, 1 error per block; random, 2 errors per block; consistent, 3 errors per block; and systematic, 4 errors/block. In the TROG-2 manual, 3 errors are also named “random” but for the current study, we defined that these errors are consistent as they are above the chance level performance. To characterize the nature of errors, we conducted within-group comparisons on the percent of two categories of errors: (1) general cognitive processing/ mild morphosyntactic dysfunction: percent of sporadic plus random errors; (2) morphosyntactic/moderate-severe deficit: percent of consistent and systematic errors.

To investigate the association between performance in the test and age, education (controls and PPA), and disease duration (PPA), we used Spearman's correlation test.

A *p*-value <0.05 was considered statistically significant and Bonferroni correction was employed to account for *post-hoc* tests.

## Results

### Subjects: Characterization

The sample consisted of 108 individuals, 74 controls (54 women), and 34 PPA (19 women) (Table 1). A chi-square test of independence was performed to examine the relationship between gender and group. Although the relationship between these variables was not significant, *X*^2^ (1, *N* = 108) = 3.106, *p* = 0.078, we observed a higher proportion of women in the control group (73%) compared to the PPA group (55.9%). The distributions of age and education in the two groups differed significantly (Mann–Whitney *U* = 699.5, *p* = 0.000 two-tailed and Mann–Whitney *U* = 529, *p* = 0.000 two-tailed, respectively), with controls being older and exposed to fewer years of formal education than the individuals with PPA. PPA subgroups (logopenic, semantic, non-fluent/agrammatic, and mixed) did not present significant differences regarding age (Kruskal–Wallis *H* = 2.568, *p* = 0.101, two-tailed) and years of formal education (Kruskal–Wallis *H* = 6.218, *p* = 0.463, two-tailed). Gender distribution was also not significantly different among the groups *X*^2^ (4, *N* =108) = 4.727, *p* = 0.316.

**Table 1:** Demographic and clinical characteristics of PPA groups and controls.

	**Sex (% Women)**	**Education (years)**		**Age**		**Disease duration (years)**	
		**Mean (SD)**	**Range**	**Mean (SD)**	**Range**	**Mean (SD)**	**Range**
Controls (*N =* 74)	73.0%	7.66 (5.12)	2.0–17.00	72.51 (7.78)	60–92	–	–
PPA (*n =* 34)	55.9%	13.24 (4.60)	4.00–21.00	66.00 (7.68)	52–81	2.41 (1.65)	0.6–7.0
PPA-L (*N =* 5)	80,0%	13.40 (3.44)	8.0–16.00	61.80 (8.07)	56–76	2.0 (1.22)	1.0–4.0
PPA-S (*N =* 12)	50.0%	15.42 (4.85)	4.0–21.00	66.33 (7.87)	52–78	2.3 (1.70)	0.6–7.0
PPA-NF/A (*N =* 6)	50.0%	11.50 (6.35)	4.0–19.00	67.83 (6.59)	60–78	2.8 (2.40)	1.0–7.0
PPA-Mx (*N =* 11)	54.5%	11.73 (2.94)	7.0–16.00	66.55 (8.21)	55–81	2.5 (1.78)	1.0–6.0

*PPA, Primary Progressive Aphasia group; PPA-L, Logopenic variant of Primary Progressive Aphasia; PPA-S, Semantic variant of Primary Progressive Aphasia; PPA-NF/A, Nonfluent/Agrammatic variant of Primary Progressive Aphasia; PPA-Mx, Mixed (unclassified) variant of Primary Progressive Aphasia. SD, Standard Deviation*.

Tables 2, 3 show the demographic, clinical, and linguistic characterization of the PPA sample. For a brief neuropsychological characterization of the control group, the Brazilian version of the revised Addenbrooke's Cognitive Examination (ACE-R) was applied. The mean ACE-R total score was 84.84 (SD = 8.82), range 67–97; Attention and orientation subscore was 16.71 (SD = 1.28), range (13–18); Fluency subscore was 10.01 (SD = 2.09), range 4–14; Language was 23.43 (SD = 3.34), range 14–26; Memory was 21.05 (SD = 3.66), range (12–26); Visuospatial was 13.62 (SD = 2.02) range 8–16; MMSE 27.47 (SD =1.93), range 23–30.

**Table 2:** Primary Progressive Aphasia subjects: Demographics, clinical characterization and neuroimaging.

**Cases**	**Ppa Variant**	**Gender**	**Age**	**Education (years)**	**Disease duration (years)**	**Neuroimaging exams**
1	PPA-L	M	59	12	2	Hypoperfusion in the left temporal cortex, more severe in the medial and inferior temporal lobe (SPECT)
2	PPA-L	F	76	8	1	Left posterior atrophy (temporo-parieto-occipital junction) (MRI)
3	PPA-L	F	60	16	4	Generalized brain atrophy, more severe in the posterior region (MRI)
4	PPA-L	F	56	15	1	Bilateral parieto-occipital atrophy (MRI)
5	PPA-L	F	58	16	2	Hypointensities in the right parieto-occipital cortex and in the left fronto-parietal cortex (MRI)
6	PPA-Mx	M	55	11	5	Generalized bilateral brain atrophy
7	PPA-Mx	M	75	15	1	Generalized bilateral atrophy, worse in the left hemisphere (MRI)
8	PPA-Mx	F	71	11	2.5	Generalized bilateral atrophy, worse in the left hemisphere (MRI)
9	PPA-Mx	M	62	10	1.6	Left fronto-temporo-parietal atrophy (MRI)
10	PPA-Mx	F	58	14	1.5	Generalized bilateral atrophy, worse in the left hemisphere (MRI)
11	PPA-Mx	F	66	11	4	Left temporo-parietal and posterior cingulate hypometabolism, extending to the left frontal lobe
12	PPA-Mx	F	81	7	6	Generalized bilateral atrophy and white matter hyperintensities (MRI)
13	PPA-Mx	F	74	15	2.6	Temporo-parietal atrophy, worse in the left hemisphere (MRI)
14	PPA-Mx	F	67	11	1	Generalized bilateral atrophy and white matter hyperintensities (MRI)
15	PPA-Mx	M	66	8	1	Bilateral medial frontal lobe hypometabolism (PET-FDG)
16	PPA-Mx	M	57	16	1	Left temporal atrophy (MRI)
17	PPA-NF/A	M	70	4	1	Left fronto-temporo-parietal atrophy (MRI)
18	PPA-NF/A	M	62	4	1	Left temporal atrophy (MRI)
19	PPA-NF/A	F	78	11	4	Left superior, medial and inferior frontal hypoperfusion (SPECT)
20	PPA-NF/A	M	66	15	3	Left fronto-temporal hypometabolism (PET-CT), anterior temporal lobe atrophy (MRI)
21	PPA-NF/A	F	60	17	1	Right insular atrophy (MRI)
22	PPA-NF/A	F	71	16	7	Left temporal atrophy (MRI)
23	PPA-S	M	66	16	3	Left temporal atrophy (MRI)
24	PPA-S	F	77	17	7	Generalized brain atrophy and bilateral hippocampal atrophy
25	PPA-S	F	70	16	3	Bilateral anterior temporal atrophy (MRI)
26	PPA-S	M	61	17	3	Left temporal atrophy (MRI)
27	PPA-S	M	65	17	2	Left fronto-temporo-parietal atrophy (MRI)
28	PPA-S	M	78	4	1	Generalized bilateral atrophy, worse in the left anterior temporal lobe (MRI)
29	PPA-S	F	52	11	2	Left anterior temporal lobe atrophy (MRI)
30	PPA-S	M	72	17	0.6	Left fronto-temporal atrophy (MRI)
31	PPA-S	F	59	11	1	Left temporal atrophy (MRI)
32	PPA-S	F	60	17	2	Generalized bilateral atrophy (MRI)
33	PPA-S	M	73	15	2	Generalized bilateral atrophy (MRI)
34	PPA-S	F	63	15	1	Left anterior temporal hypoperfusion (SPECT)

*PPA-L, Logopenic variant of Primary Progressive Aphasia; PPA-S, Semantic variant of Primary Progressive Aphasia; PPA-NF/A, Nonfluent/Agrammatic variant of Primary Progressive Aphasia; PPA-Mx, Mixed (unclassified) variant of Primary Progressive Aphasia. SD, Standard Deviation. M, Male, F, Female*.

**Table 3:** Primary Progressive Aphasia subjects: Language Assessment.

**Cases**	**Ppa Variant**	**Fluency**		**Naming**		**Oral agility**		**Repetition**		**Auditory comprehension**			**Oral Reading and reading comprehension**		
		**SVF**	**LVF**	**BNT**	**CNT**	**BNVA**	**BVA**	**BRW**	**BSR**	**CWC**	**BC**	**BICM**	**BWR**	**BSR**	**BSPRC**
				**(Max. 60)**	**(Max.64)**	**(Max.12)**	**(Max.14)**	**(Max.10)**	**(Max;16)**	**(Max.64)**	**(Max.15)**	**(Max.12)**	**(Max.30)**	**(Max.10)**	**(Max.10)**
1	PPA-L	3	15	NA	29	7	14	0	0	59	NA	NA	NA	NA	NA
2	PPA-L	10	13	40	56	7	14	10	11	56	9	7	29	8	6
3	PPA-L	14	21	52	63	11	10	6	6	63	13	10	29	10	10
4	PPA-L	13	33	41	57	12	13	10	12	63	14	9	30	10	10
5	PPA-L	8	16	30	53	5	6	10	4	55	3	2	30	7	8
6	PPA-Mx	4	1	19	37	NA	7	7	2	47	NA	NA	9	5	0
7	PPA-Mx	8	5	5	NA	NA	5	1	1	55	4	NA	29	8	NA
8	PPA-Mx	6	8	15	24	12	9	8	2	55	3	4	28	8	5
9	PPA-Mx	2	0	14	21	10	12	6	0	48	2	0	4	0	0
10	PPA-Mx	5	8	16	NA	0	0	10	9	NA	10	3	30	10	8
11	PPA-Mx	3	8	6	22	7	12	10	1	43	7	6	30	10	5
12	PPA-Mx	4	5	12	26	11	13	10	11	59	13	1	30	10	7
13	PPA-Mx	10	24	25	58	11	7	8	3	58	10	8	30	7	7
14	PPA-Mx	18	26	41	61	11	10	10	14	64	14	8	30	10	9
15	PPA-Mx	10	12	33	61	6	6	10	14	61	14	9	30	10	8
16	PPA-Mx	8	16	48	58	9	12	10	15	64	15	11	30	10	10
17	PPA-NF/A	3	3	21	43	9	10	7	1	53	NA	NA	0	0	0
18	PPA-NF/A	1	0	NA	0	4	10	2	0	51	11	4	0	0	4
19	PPA-NF/A	5	9	33	55	10	12	9	10	55	11	6	24	9	4
20	PPA-NF/A	5	2	35	52	10	1	9	10	61	11	8	30	7	9
21	PPA-NF/A	17	27	41	61	3	2	10	10	61	14	6	30	10	9
22	PPA-NF/A	9	10	52	63	0	0	7	6	64	12	12	30	10	10
23	PPA-S	0	0	1	2	12	14	10	11	39	0	0	30	10	NA
24	PPA-S	5	6	8	0	10	13	8	9	44	9	1	24	9	5
25	PPA-S	3	1	8	26	12	13	10	4	58	7	6	27	8	6
26	PPA-S	8	21	25	47	10	10	9	12	60	15	8	30	10	9
27	PPA-S	10	13	28	51	6	5	9	8	60	12	8	30	10	9
28	PPA-S	9	2	16	34	0	0	10	12	48	12	9	30	9	5
29	PPA-S	9	32	16	30	10	10	10	13	59	13	6	29	10	4
30	PPA-S	4	29	14	27	12	14	NA	NA	59	NA	NA	30	10	NA
31	PPA-S	10	16	29	52	10	13	10	0	62	15	8	30	10	9
32	PPA-S	14	27	30	51	10	14	10	16	60	14	10	30	10	9
33	PPA-S	2	23	17	27	10	10	10	14	50	11	8	30	10	9
34	PPA-S	10	33	42	57	10	14	10	16	64	13	11	30	10	9

*SVF, semantic verbal fluency (animals/min); LVF, letter verbal fluency [(F+A+S)/min]; BNT, Boston naming test; Subtests of the Boston Diagnostic Aphasia Evaluation, BC, Auditory Comprehension (Commands) BCIM, Auditory Comprehension (Complex Ideational Material); BNVA, Non-verbal agility (max. 12), BVA, Verbal Agility (max.14); BRW, repetition of words; BSR, sentence repetition (sum of low and high frequency sentences), BWR, Oral reading of words, BSR, Boston oral reading of sentences, BSPRC, sentences and paragraphs reading comprehension; subtests of the Cambridge Semantic Memory Research Battery (CSMRB) (55–57): CWC, word comprehension; CNT, Cambridge naming test; NA, Non applied/available; PPA-L, Logopenic variant of Primary Progressive Aphasia; PPA-S, Semantic variant of Primary Progressive Aphasia; PPA-NF/A, Nonfluent/Agrammatic variant of Primary Progressive Aphasia; PPA-Mx, Mixed (unclassified) variant of Primary Progressive Aphasia. Max, Maximum score*.

### Influence of Demographic and Clinical Characteristics of Controls and PPA Subjects on TROG2-Br

Pearson's chi-square test of independence was performed to examine the relationship between gender and the number of blocks passed. We did not find significant differences associated with gender neither in the control group [*X*^2^ (15, *N* = 74) = 15.463, *p* = 0.419] nor in the PPA group [*X*^2^ (17, *N* =34) = 14.202, *p* = 0.653].

In the control group, we found a significant positive correlation between the number of passed blocks and years of formal education: Spearman's *r* = 0.33, *p* = 0.004. The correlation between age and the number of passed blocks was not significant, Spearman's *r* = 0.06, *p* = 0.579.

In the PPA group, the correlations between the number of correct blocks and education, age, and disease duration were not significant: *r* = 0.27, *p* = 0.120; *r* = −16 *p* = 0.358; *r* = −0.30, *p* = 0.08, respectively.

### Performance of Controls and PPA on TROG2-Br

**Figure 1:**
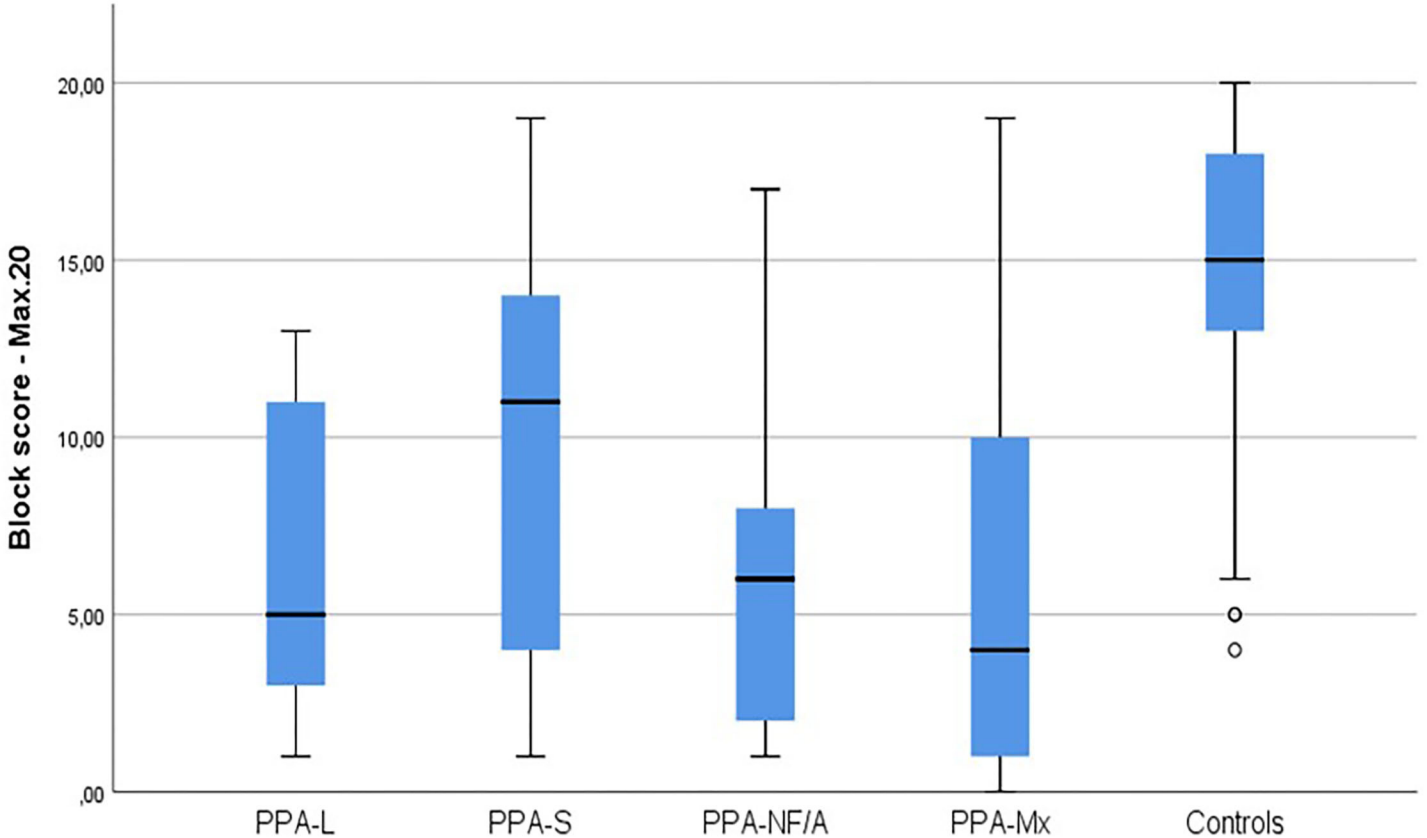
Performance of PPA subjects and controls in the TROG2-Br. PPA, Primary Progressive Aphasia group; PPA-L, Logopenic variant of Primary Progressive Aphasia; PPA-S, Semantic variant of Primary Progressive Aphasia; PPA-NF/A, Nonfluent/Agrammatic variant of Primary Progressive Aphasia; PPA-Mx, Mixed (unclassified) variant of Primary Progressive Aphasia. Max20, Maximum score is 20 blocks.

Table 4 and Figure 1 present the results of controls and PPA on TROG2-Br: overall score on blocks and items, types of errors (sporadic, random, consistent, and systematic), and categories of errors (general processing or morphosyntactic). Controls presented a higher number of correct responses (Mann–Whitney *U* = 334.5, *p* =0.000, two-tailed) and passed blocks (Mann–Whitney *U* = 402.0, *p* =0.000, two-tailed) compared to PPA. The median of correct blocks in the control group was 15 and the scores for the 10, 25, 75, and 90 percentiles were, respectively, 10, 13, 18, and 20 blocks. Most PPA patients presented scores below the median of controls (*n* = 30). The patients with more preserved sentence comprehension (median or above compared to controls) were cases 16 (PPA-Mx); 22 (PPA-NF/A); and 33 and 34 (both PPA-S). Comparing the four PPA subgroups, we found no significant differences in the performance both considering the correct items (Kruskal-Wallis H=3.918, *p* =0.270, two-tailed) and the number of passed blocks (Kruskal–Wallis *H* = 2.724, *p* = 0.436, two-tailed).

**Table 4:** Performance of the control and PPA groups in TROG2-B—Overall accuracy, types and categories of errors.

	**Overall Accuracy**						
	**Group**	**N**	**Mean**	**SD**	**Range**	Mann-Whitney U*	**p**
Correct items (*N =* 80)	CG	74	73.58	6.05	51–80	334.50	<0.0000
	PPA	34	52.91	16.65	26–79		
	PPA-L	5	52.20	13.08	39–68		
	PPA-S	12	60.33	15.22	36–79		
	PPA-NF/A	6	50.00	15.75	30–76		
	PPA-Mx	11	46.73	18.83	26–79		
Correct blocks (*N =* 20)	CG	74	15.04	3.77	4–20	402.00	<0.000
	PPA	34	7.56	5.96	0–19		
	PPA-L	5	6.60	5.18	1–13		
	PPA-S	12	9.75	5.99	1–19		
	PPA-NF/A	6	6.67	5.72	1–17		
	PPA-Mx	11	6.09	6.44	0–19		
	**Types of errors**						
	**Group**	**N**	**Mean**	**SD**	**Range**	**Mann-Whitney U**	**p**
% of sporadic errors (1/block)	CG	66	73	28.28	0–73	310.00	<0.000
	PPA	34	27	28	2–100		
	PPA-L	5	17	6	7–25		
	PPA-S	12	40	36	8–100		
	PPA-NF/A	6	17	17	4–50		
	PPA-Mx	11	23	28	2–100		
% of random errors (2/block)	CG	66	22	24.45	0–24	860.00	0.052
	PPA	34	30	18	0–83		
	PPA-L	5	43	25	19–83		
	PPA-S	12	28	19	0–67		
	PPA-NF/A	6	37	11	24–52		
	PPA-Mx	11	22	14	0–42		
% of consistent errors (3/block)	CG	66	5	12.31	0–12	433.50	<0.000
	PPA	34	28	21	0–75		
	PPA-L	5	24	22	0–44		
	PPA-S	12	23	24	0–75		
	PPA-NF/A	6	31	22	0–60		
	PPA-Mx	11	34	18	0–61		
% of systematic errors (4/block)	CG	66	1	3.27	0–3	534.5	<0.000
	PPA	34	15	18	0–81		
	PPA-L	5	16	11	0–25		
	PPA-S	12	9	12	0–34		
	PPA-NF/A	6	14	19	0–40		
	PPA-Mx	11	21	25	0–81		
	**Categories of Errors**						
	**Group**	**N**	**Mean**	**SD**	**Range**	**Mann-Whitney U**	**p**
% Processing errors	CG	66	95	13.55	47–95	343.50	<0.000
	PPA	34	57	28	13–100		
	PPA-L	5	60	27	37–100		
	PPA-S	12	69	29	25–100		
	PPA-NF/A	6	55	25	36–100		
	PPA-Mx	11	45	26	13–100		
% Morphosyntactic errors	CG	66	5	13.55	0–14	343.50	<0.000
	PPA	34	43	28	0–87		
	PPA-L	5	40	27	0–63		
	PPA-S	12	31	29	0–75		
	PPA-NF/A	6	45	25	0–64		
	PPA-Mx	11	55	26	0–87		

PPA, Primary Progressive Aphasia group; PPA-L, Logopenic variant of Primary Progressive Aphasia; PPA-S, Semantic variant of Primary Progressive Aphasia; PPA-NF/A, Nonfluent/Agrammatic variant of Primary Progressive Aphasia; PPA-Mx, Mixed (unclassified) variant of Primary Progressive Aphasia. SD, Standard Deviation. *Mann-Whitney U and p = comparisons between PPA group and Control group. 8 controls did not present errors, so for error analysis the number of controls is 66.

Eight controls presented 100% accuracy, so for error analysis, the total number of control participants is 66. No PPA patient scored 100%. The analysis of errors evidenced that the control group had a higher proportion of sporadic errors (*p* < 0.0001) and a lower proportion of consistent and systematic errors (*p* < 0.0001) compared to the PPA and PPA subtypes. The percent of random errors was not significantly different between the controls and the other groups (*p* = 0.860 in the comparison with PPA; *p* = 0.284; *p* = 0.780; *p* = 0.07; *p* = 0.05 in comparisons with PPA-S, PPA-Mx, PPA-L, and PPA-NF/A, respectively). In the comparisons between PPA subtypes, the differences were not statistically different, although there was a trend toward a different proportion of processing vs. morphosyntactic errors in PPA-S compared to PPA-Mx (*p* = 0.07); in PPA-Mx, errors were similarly distributed in both categories, whereas PPA-S had more processing than morphosyntactic errors and between PPA-NF/A and PPA-Mx in random errors (*p* = 0.045), the proportion was higher in PPA-NF/A (refer to Table 4). Within-group comparisons pointed to a greater proportion of processing (sporadic + random errors) than morphosyntactic (consistent + systematic errors) in the control group (Wilcoxon *Z* = −7.65; *p* < 0.0001) and a similar profile although only marginally significant for PPA-S (Wilcoxon *Z* = −1.706; *p* = 0.08). In the other groups, the proportion of processing vs. morphosyntactic errors was not significantly different: PPA-NF/A (Wilcoxon *Z* = −0.105; *p* = 0.917); PPA-L (Wilcoxon *Z* = −0.730; *p* = 0.465), and PPA-Mx (Wilcoxon *Z* = −0.764; *p* = 0.445).

### Types of Errors and Blocks Where Errors Occurred

Tables 5, 6 present the performance of controls and patients with PPA on each block, according to the number of errors (0 = passed block, 1, 2, 3, or 4 errors per block). The individual performance of patients with PPA is available in the Supplementary Materials. Across all blocks, a higher percentage of controls (more than 60%) passes the block (zero errors), followed by a percentage that makes a sporadic error (1 error). Few controls make more than one error and only one control (1.4%) makes 4 errors in the same block (blocks J and T). Sporadic and random errors occur even in simpler blocks (A, B, and C) both in the control and PPA groups.

**Table 5:** Performance of individuals in the control group in each block (grammatical and syntactic structures assessed by TROG-2). Results refer to the percentage of individuals who passed or failed the blocks and their number of errors (1,2,3 our 4), (*n* = 74).

**Block**	**Passed % of individuals**	**1 error % of individuals**	**2 errors % of individuals**	**3 errors % of individuals**	**4 errors % of individuals**	**Structures**	**Examples**
A	79.7	14.9	4.1	1.4		Two elements	The sheep is running
B	75.7	17.6	5.4	1.4		Negative	The man is not sitting
C	60.8	36.5	2.7			Reversible in and on	The cup is in the box
D	87.8	12.2				Three elements	The girl pushes the box
E	87.8	10.8	1.4			Reversible SVO	The cat is looking at the boy
F	71.6	25.7	2.7			Four elements	The horse sees the cup and the book
G	75.7	16.2	6.8	1.4		Relative clause in subject	The man that is eating is looking at the cat
H	68.9	29.7	1.4			Not only X but also Y	The pencil is not only long but also red
I	77.0	18.9	4.1			Reversible above and below	The flower is above the duck
J	71.6	18.9	4.1	4.1	1.4	Comparative/absolute	The duck is bigger than the ball
K	64.9	23.0	10.8	1.4		Reversible passive	The cow is chased by the girl
L	71.6	16.2	9.5	2.7		Zero anaphor	The man is looking at the horse and he is running
M	75.7	16.2	6.8	1.4		Pronoun gender/number	They are carrying him
N	77	20.3	1.4	1.4		Pronoun binding	The man sees that the boy is pointing at him
O	85.1	12.2	2.7			Neither nor	The girl is neither pointing nor running
P	90.5	5.4	4.1			X but not Y	The cup but not the fork is red
Q	75.7	14.9	8.1	1.4		Post modified subject	The elephant pushing the boy is big
R	78.4	18.9	2.7			Singular/plural inflection	The cows are under the three
S	66.2	29.7	4.1		1.4	Relative clause in object	The girl chases the dog that is jumping
T	60.8	24.3	8.1	5.4		Center-embedded sentence	The sheep the girl looks at is running

**Table 6:** Performance of individuals in the PPA group in each block (grammatical and syntactic structures assessed by TROG-2 and TROG2-Br). Results refer to the percentage of individuals who passed or failed the blocks and their number of errors (1,2,3 our 4) (*n* = 34).

**Block**	**Passed % of individuals**	**1 error % of individuals**	**2 errors % of individuals**	**3 errors % of individuals**	**4 errors % of individuals**	**Structures**	**Examples**
A	73.5	26.5				Two elements	The sheep is running
B	67.6	14.7	11.8	2.9	2.9	Negative	The man is not sitting
C	29.4	47.1	14.7	8.8		Reversible in and on	The cup is in the box
D	79.4	8.8	11.8			Three elements	The girl pushes the box
E	47.1	32.4	14.7	5.9		Reversible SVO	The cat is looking at the boy
F	50	20.6	14.7	11.8	2.9	Four elements	The horse sees the cup and the book
G	41.2	8.8	29.4	14.7	5.9	Relative clause in subject	The man that is eating is looking at the cat
H	44.1	26.5	8.8	14.7	5.9	Not only X but also Y	The pencil is not only long but also red
I	38.2	26.5	23.5	11.8		Reversible above and below	The flower is above the duck
J	47.1	8.8	8.8	23.5	11.8	Comparative/absolute	The duck is bigger than the ball
K	11.8	11.8	38.2	26.5	11.8	Reversible passive	The cow is chased by the girl
L	17.6	11.8	32.4	20.6	17.6	Zero anaphor	The man is looking at the horse and he is running
M	32.4	17.6	32.4	8.8	8.8	Pronoun gender/number	They are carrying him
N	32.4	17.6	26.5	17.6	5.9	Pronoun binding	The man sees that the boy is pointing at him
O	47.1	11.8	14.7	17.6	8.8	Neither nor	The girl is neither pointing nor running
P	32.4	25.5	11.8	20.6	8.8	X but not Y	The cup but not the fork is red
Q	17.6	26.5	29.4	17.6	8.8	Post modified subject	The elephant pushing the boy is big
R	29.4	20.6	32.4	14.7	2.9	Singular/plural inflection	The cows are under the three
S	17.6	8.8	17.6	41.2	14.7	Relative clause in object	The girl chases the dog that is jumping
T	5.9	5.9	23.5	32.4	32.4	Center-embedded sentence	The sheep the girl looks at is running

Regarding patients with PPA, the performance on TROG2-Br was qualitatively different from controls. In 3 out of 20 blocks, most patients make no errors (A, B, and D) and 50% of PPA individuals pass block F. Blocks A, D, and F increase the number of elements but not syntactic complexity (two, three, and four elements, respectively). Block B tests negative sentences. On the other blocks, performance is more varied but on blocks S and T, most patients make 3 or 4 errors, which is considered a consistent or systematic error. These blocks contain noncanonical sentences, object relative clauses, and center-embedded sentences.

### Internal Consistency and Concurrent Validity of TROG2-Br

The value for Cronbach's alpha was α = 0.87, which is considered a good internal consistency for test (68). It was computed for the score of 20 blocks for a subsample of 94 participants (those who obtained a minimum score of 5 correct blocks, 73 controls and 21 PPA). All controls undertook TROG2-Br and the Brazilian short version of Token Test (*n* = 74). In addition, 26 patients with PPA were also evaluated with both tests. There was a positive significance and high correlation between the percentage of correct responses on TROG2-Br and the validated Brazilian version of the Token Test (Spearman's *r* = 0.765, *p* < 0.000), indicating a good concurrent validity.

### A Suggestion of a Shorter Version of TROG2-Br

We used Cronbach's alpha as a parameter to create a shorter version of TROG2-Br, which may be useful to test populations with limited sustained attention or in contexts of time constraints. For this purpose, our sample was composed of 21 PPA and 73 controls, as mentioned before. Fifteen blocks were excluded in the following order in successive iterations, from those blocks contributing less to the internal consistency of the test to those contributing more: D, B, A, H, F, C, O, E, R, G-J- S-P-N-I. The internal consistency of the five suggested blocks was α = 0.82 (blocks T-M-Q- K- L). The syntactic structures evaluated by these blocks are described in Tables 5, 6.

## Discussion

Sentence comprehension is a core domain to be investigated in patients with brain injuries and particularly in patients with PPA, as this ability is a supplementary criterion for the classification of PPA variants (6). There is a paucity of tools to evaluate this domain in more depth. Moreover, few studies characterize populations with heterogeneous exposure to formal education and populations that use other languages than English.

Grammar and syntax can be evaluated through reception and production tasks, offline, or online (see Wilson et al. (9, 14); Grossman (69); Thompson and Mack (18); Mesulam (10) for comprehensive reviews). The latter poses less impact on generalized cognitive resources and is more appropriate to investigate the neural correlates of sentence processing (14, 24, 70, 71). Offline tasks are often used in clinical settings and are more available to neuropsychologists and speech and language therapists that are directly involved in planning and executing interventions for PPA individuals. In PPA, most studies focused on measures of connected speech to detect agrammatism (13–17, 42, 72, 73) (Thompson and Mack (18), Boschi et al. (19) for reviews), which is a core feature of APP-NF/A (10, 21, 25, 69). Although these tasks have been considered the gold standard for this purpose, analyzing connected speech is not always practical in clinical contexts (74). Compared to production, morphosyntactic processing in reception tasks across PPA subtypes is less reported. One reason for that may be that these tasks have shown a considerable overlap in the overall accuracy measures between the three subtypes of PPA or even between PPA-S and PPA-NF/A (3, 23, 27, 74, 75), similar to the findings of the current study. Sentence comprehension and production recruit a frontotemporal network bilaterally and, while the former ability engages more regions in the right hemisphere, the latter is more left-lateralized (refer to Walenski et al. (39), for a comprehensive review and meta-analysis). Therefore, whereas sentence production is likely to be more selectively impaired in PPA-NF/A, sentence comprehension can be impaired due to lesions affecting a more widespread neural network and reflecting processes beyond morphosyntactic deficits, such as lexical-semantic, working memory, and executive dysfunction. That said, a comprehensive sentence comprehension assessment should not only be undertaken for subclassification purposes but also for monitoring symptom progression and designing tailor-made interventions to improve the communication and quality of life for individuals suffering from PPA. Thus, the first contribution of this study is to introduce and demonstrate the applicability of the Brazilian version of TROG-2 for Portuguese speakers, a well-designed and comprehensive tool to investigate sentence comprehension in clinical settings.

Although TROG was initially devised to investigate grammar and syntactic development in children (76), it has been validated for the assessment of comprehension in the sentence level in populations of children and adults (40). It is a comprehensive task that allows for the detection of a more generalized comprehension disorder, as well as for the identification of impairments to process specific grammar contrasts and syntactic structures. Portuguese is one of the most spoken languages in the world. It is characterized by a rich morphology and syntax and, to the best of our knowledge, there are no validated instruments to assess a wide range of morphosyntactic contrasts in adults. In the present study, we describe the procedures for translation and adaptation of TROG-2 and introduce TROG2-Br for the assessment of auditory sentence comprehension in typical aging and in patients with PPA. We evaluate its internal consistency and concurrent validity against the Brazilian short version of the Token Test, which has been validated for the assessment of older adults in a similar population (37). Additionally, we investigate the association between performance in TROG2-Br and age and years of formal education for the whole sample and disease duration in the PPA group. Another objective was to characterize the performance of older adults and of a sample of individuals with PPA with PPA-S, PPA-NF/A, PPA-L, and PPA-Mx phenotypes, speakers of Portuguese, evaluating quantitative and qualitative aspects of performance.

The Brazilian Version of the Test for the Reception of Grammar keeps the basic properties of TROG-2 (number of blocks and stimuli) and is applied using the same stimuli book as that of TROG-2. It has shown good internal consistency and concurrent validity, demonstrating that the tool is evaluating the targeted domain by a correlation among its component blocks and with a validated task of sentence comprehension. In the following sections, we discuss the performance of typical older adults with heterogeneous levels of education in TROG2-Br, the findings with PPA patients, and make considerations about the use of TROG2-Br and future directions of research with this tool.

### Performance of Community-Dwelling Adults (Typical Aging) and PPA on TROG2-Br

Previous studies with TROG, conducted in samples with higher years of formal education, reported ceiling effects (27, 42, 74, 75, 77, 78). Differently, this study included individuals with different educational levels and evidenced a wider range of scores and median performance of 15 blocks (refer to Table 4). Educational level, socioeconomic status, vocabulary size, and reading and writing habits are factors that may influence language comprehension (and other cognitive functions) in the elderly (54, 79–86). In the present study, education was positively correlated to accuracy on TROG2-Br corroborating findings of previous studies and suggesting that low educational level, associated with aging, may potentiate the risks for language decline (81).

Elucidating the role of schooling in sentence comprehension in elders was directly targeted in a study with 405 Brazilian Portuguese speakers (83). Sixty-nine percent of the sample population had low scores in the Token Test, with 13% classified as severely impaired. The severity of failure was positively associated with age and schooling; thus, it was not possible to discriminate the relative weight of each factor. Later, in consideration of the influence of schooling, normative scores for the elderly in the Token Test were proposed (37). Further evidence for the influence of schooling on the Brazilian population was gathered in a recent study that analyzed the performance of 111 cognitively healthy elders in the Revised Token Test (81). The authors observed that the group of low schooling presented fewer hits than the group of high schooling in all blocks of the test. Although the Revised Token Test bears some differences from the TROG2-Br, the results of both tests support the view of the impact of education on sentence comprehension. However, this finding should be taken with a degree of caution, given that the quality of education varies widely in Brazil (87, 88). Additionally, studies on participants from other countries have shown that differences in the quality of education, when measured by reading, writing, and cultural skills, contribute to differences in performance in cognitive tests (89–91).

It is important to notice, however, that with few exceptions, the general response pattern of older adults in TROG2-Br was sporadic errors, characterized as giving an incorrect answer to only one sentence but answering correctly the three other sentences of the block (refer to Table 5). The occurrence of this phenomenon is suggestive of processing difficulties rather than genuine morphosyntactic deficits and is consistent with the literature on language in healthy aging [Wingfield and Stine (92); Argimon and Stein (93); Yun and Lachman (94)]. Most sporadic errors occurred in blocks demanding syntactic processing and working memory resources (i.e., blocks S and T). Similarly, in the standardization study in the UK, Block T presented the highest number of errors in aged adults, presumably because it requires more working memory resources, in terms of sentence length and syntactic operations. Bishop (40) pointed out that sporadic errors in block T are expected to occur among cognitively healthy adults. A finding that requires further exploration is the occurrence of errors in Blocks C and H, in which we observed sporadic errors in 36.5 and 29.7% of our sample, respectively, and that may suggest difficulties to manipulate visuoperceptual and visuospatial information for comprehension in older adults. In addition, Blocks A and B should not pose any difficulty for cognitively healthy adults; however, some individuals presented errors. Errors in Block A in individuals who demonstrated a high overall accuracy score suggest that instructions, examples, and training should be maximized in the further use of this task with older adults, especially for low-educated individuals. Negative sentences (Block B) require more time for processing as in formal tests, which are presented in a context that rarely occurs in daily life, thus resulting in an increased error rate (95). In oral language, negative sentences are typically used when the proposition that was mentioned earlier needs corrections and then, the speaker intends to communicate deviations from what has been said (96, 97). In this regard, TROG-2 presents an infrequent situation and may increase the number of errors in individuals with lower levels of education who might be less familiar with formal testing situations.

In several studies, gender was not found to play a major role in sentence comprehension (37, 40, 81, 83) and in other language tasks, such as BNT (98). In the same direction, in the current study, we found similar performance for men and women. However, as the sample was composed predominantly of women, conclusions regarding the effect of gender are limited.

The block score of TROG2-Br did not present a significant correlation with age. Our findings corroborate other clinical studies (37, 54, 99, 100) but are different from a previous study (82) on the Brazilian Portuguese investigating sentence comprehension with the Token Test and in a wider aging range (50–80 years old). In that study, the authors used a scoring system that considered both accuracy and execution time. The latter variable was more sensitive to detecting age-related changes whereas accuracy was similar among all age groups. Carvalho et al. (83) also found an association between age and performance (accuracy) in the Token Test; however, in that study, the older individuals were also less exposed to formal education compared to the younger groups as mentioned previously. The Token Test requires the execution of commands of different lengths and poses high demands on working memory (82). In the normative study of TROG-2 (40), the sample of aged individuals ranged from 65 to 86 years, similar to the age range in the current study. Alike our results, in the UK standardization study, age did not have an impact on the task. The standardization of the elderly sample had similar scores to the young and adolescents aging 14 years and above. These data suggest that the ability to understand literal sentences does not tend to diminish markedly with the advance of age, as proposed for other aspects of cognition (93, 94, 101). The relationship between TROG2-Br and other neuropsychological variables as well as the response times remain to be explored in future studies. Moreover, the need for education-adjusted scores should also be investigated for wider use of the test in clinical settings.

In sum, our results support the findings of previous studies and claim that working memory and/or processing speed impact the ability to comprehend more complex structures instead of a syntactic deficit associated with healthy aging (92, 102). Comprehension of sentences with syntactic movements requires lexical-semantic retrieval, working memory, and attentional processes. Consequently, in situations of cognitive overload, auditory comprehension can be altered in the elderly with no direct relation to reduced linguistic cognitive abilities. An example of such a situation would be when an elder listens to an extremely long text, with noncanonical syntactic structures, or to a very fast-speed speech (92, 103, 104).

### Performance of Patients With PPA and Differentiation of PPA Subtypes

Despite having more years of formal education and being younger than the control group, patients with PPA presented significantly impaired performance on TROG2-Br compared to the controls. Not only the accuracy was lower (30 out of 34 patients presented scores below the median of controls) but also the errors were qualitatively different, with a greater proportion of 3 or 4 errors at the same block, pointing to difficulties or total inability to process specific grammar contrasts and syntactic structures. Our results corroborate previous findings with TROG in different samples of PPA and frontotemporal dementia syndromes (27, 41, 42, 58–60, 74, 75, 77, 78) that consistently demonstrated a difference between controls and patients with PPA. As controls presented mostly sporadic errors and higher scores in TROG2-Br, this tool may be useful for the detection of a receptive morphosyntactic deficit and to characterize the sentence structures in which PPA individuals have difficulties. It is important to mention that education, age, and disease duration were not associated with performance in the PPA group, pointing to a major role of morphosyntactic processing difficulties rather than other factors as an explanation for these findings.

Comparative studies of grammatical comprehension in PPA variants are rare (23). In the present study, performance was highly heterogeneous within PPA subtypes. Apart from PPA-L, in which all patients performed below the median of controls, the other three groups presented individuals with high scores, at the same levels as highly performing controls. Although the comparisons among PPA subtypes did not reach statistical differences in our sample, performance patterns across groups were compatible with previous studies, in which PPA-S presented higher scores, followed by PPA-NF/A, PPA-L, and PPA-Mx.

Sentence comprehension is usually spared in PPA-S (21, 25) (refer to Mesulam et al. (10); Wilson et al. (9); Thompson and Mack (18) for reviews). However, in the current study, PPA-S as a group presented lower scores in sentence comprehension tasks compared to controls and this has been a consistent finding across studies (3, 23, 27, 74, 75, 77). Looking more specifically at the morphosyntactic aspects of sentence comprehension, patients with PPA-S were found to be more preserved in noncanonical, monopropositional, and multiclausal relative sentences compared to the other PPA variants (3), and similarly impaired at center-embedded sentences, involving greater cognitive resource demands (23). Lack of statistical differences in the comparisons between PPA-NF/A and PPA-S in the TROG and/or similar offline tasks of sentence comprehension was also found in previous research (3, 8, 23, 27, 74, 75, 77, 78) (refer to Wilson et al. (9, 14) for reviews). In fact, sentence comprehension can be impaired in the progression of PPA-S, although semantic dysfunction is always the prominent deficit in these patients (21, 43, 70) (refer to Thompson and Mack (18) for a review). In a longitudinal study, Cupit et al. (105) found significant differences in TROG performance between APP-NF/A and APP-S in the first assessment but not on follow-up, demonstrating the decline of PPA-S in this ability as the disease progresses. Therefore, the lack of differentiation in overall scores between PPA subgroups may reflect the progression of disease in some patients with PPA-S. The underlying reason for failing the task differs among these subgroups (8, 71). In our sample, only PPA-S patients presented a marginally significantly lower percentage of consistent and systematic errors compared to sporadic and random, alike controls and compatible with general processing difficulties rather than a genuine morphosyntactic deficit. Graham et al. (27) reported a higher percentage of patients with PPA-S performing in the control range compared to other variants. We did not replicate this finding, but our sample of PPA-L and PPA-NF/S is very small, so conclusions are limited and need to be explored in larger samples. Different symptomatologies in PPA align with the degree of neurodegeneration in the language network of the left hemisphere (10). The authors explain that sentence comprehension can be maintained if the meanings of nouns of the sentence can be retrieved even at a generic level. In a few cases, PPA-S may present a sentence-comprehension deficit similar to Wernicke aphasia, but with preserved repetition because the superior temporal gyrus and temporoparietal junction are spared, and these areas exert top-down modulation to the anterior temporal lobe for comprehension (10).

Another interesting finding was the preservation of sentence comprehension in PPA-NF/A in one of the patients in our sample. This was reported in another study that used TROG (27). The authors also investigated language production in the same patients and found some PPA-NF/A individuals without frank agrammatism. They stated that this feature does not preclude a PPA-NF/A diagnosis and that differentiation between PPA-L and PPA-NF/A may be hard in some cases. In fact, agrammatism is more evident in production when the disease is very mild and may be evidenced only in writing expression in some patients (10). Thompson et al. (22) reported the need for linguistically sophisticated tools to evaluate agrammatism in PPA. The authors have worked on several measures that can be used in the clinical context (22, 106) and that have been successful in differentiating PPA subtypes. Billette et al. (74) also developed a task for sentence production that does not require the analysis of connected speech and suggested that this procedure is more applicable than the connected speech for clinical practice. However, these studies have been conducted mostly in samples of highly educated individuals and English speakers, so it is necessary to explore these features in more diverse populations. In fact, a more language-diverse assessment is a necessity in the research on dementia (107).

Except for case 22, all PPA-NF/A presented deficits in sentence comprehension. This is a more common presentation for this subgroup of patients, evidencing a two-way deficit (decoding and encoding difficulties) often reported in this clinical syndrome (10), attributed to atrophy in the inferior frontal gyrus (IFG), considered a critical hub for morphology, syntax, and grammar comprehension (108–110) (refer to Mesulam et al. (10); Walenski et al. (39) for a review). PPA-NF/A has difficulties in the comprehension of grammatically complex sentences, that include subordinate and embedded sentences (13, 20, 21, 25). The difficulties in this variant are influenced by the grammatical complexity of the sentence, different from PPA-L where the deficits are related to phonological short-term memory and affected by sentence length and frequency (predictability of the upcoming elements of a sentence) (6, 25).

Comparisons between PPA-NF/A and PPA-L in previous studies are controversial. Whereas, Thompson et al. (22) found similar performances in these groups, with a trend for more severe impairment in noncanonical sentences in PPA-NF/A and no differences between groups in the comprehension of canonical forms, others have shown lower scores in PPA-L than PPA-NF/A (23, 27), similar to our findings. Working memory has a crucial role in auditory sentence comprehension (111, 112). As phonological short-term memory is the core deficit in PPA-L (25), it is expected a high impact of this deficit in tasks, such as TROG-2 that contain many long sentences with more than two propositions. PPA-L has shown a length effect in the comprehension of canonical and noncanonical sentences with worse performance for longer than shorter items (13) (refer to Wilson et al. (9, 14); Mesulam et al. (10); Thompson and Mack (18) for reviews).

A recent study (24) analyzed online sentence comprehension in PPA-NF/A and PPA-L using event-related potentials (ERP) recorded during semantic, morphosyntactic, and verb-argument violations. In the above experiment, PPA-NF/A and PPA-L were impaired compared to controls in all conditions but no significant difference in accuracy was found between PPA-NF/A and PPA-L. However, ERPs differentiated PPA-NF/A from PPA-L. The N400 was elicited as in controls for semantic violations in both groups. On the other hand, the P600 component was not elicited in PPA-NF/A patients both for the morphosyntactic violations (e.g., The actors *was*…) and verb argument violations (e.g., Ryan was devouring *on the couch*). In PPA-L, morphosyntactic violations elicited a P600, but not verb argument violations. These findings support the different nature of deficits in PPA-NF/A and PPA-L and highlight the importance of more studies using online measures in combination with techniques, such as EEG and eye-tracker to investigate the language in PPA.

Finally, PPA-Mx presented the lower scores in our sample which is in line with previous studies with TROG and similar tasks. Billette et al. (74) found a significant difference between PPA-Mx and PPA-S with greater deficits in PPA-Mx. Sajjadii et al. (77) found that PPA-Mx performance was lower than PPA-L and PPA-NF/A in one of the sentence comprehension tasks used in their study. Although in most studies, PPA-Mx refers to a more impaired group [e.g., Billette et al. (74)], this should not be generalized. For example, in our sample, one PPA-Mx presented with very mild semantic and working memory deficits and did not meet the criteria either for PPA-S or PPA-L. This patient was also preserved in TROG2-Br. Language and neuropsychological heterogeneity in PPA has been evidenced in previous studies (29, 30, 113).

In studies combining sentence-comprehension assessment and neuroimaging findings, the deficit in PPA-NF/A was related to atrophy in the left (IFG) (8, 13, 114). This region has an important role in sentence comprehension both in the grammatical processing of long-distance dependencies between words in a sentence (115) and working memory to retain the sentence for online processing (116). The findings of Cooke et al. (114) and Peelle et al. (8) with PPA-NF/A and the behavioral variant of frontotemporal dementia support a functional dissociation in IFG, in which the dorsal parts are related to working memory demands and the ventral, with grammatical complexity.

### Final Considerations

Test for the Reception of Grammar (and TROG2-Br) requires at least 15 to 20 min to be applied in high performing or severely impaired subjects, in which the test is discontinued after 5 failed blocks. As neuropsychological and language assessment usually comprises the evaluation of other functions and subdomains; it is not always feasible to implement the full task both due to time constraints and cognitive demands over attentional processes. For these reasons, previous studies opted for shorter versions (42, 74, 75, 77, 78). The high occurrence of sporadic errors both among controls and PPA in our sample is an indicator of the demand for general cognitive resources that may be related to the duration of the task. For this reason, we obtained the internal consistency for a shorter version of the test to be tested in a similar population and compared it with the full version in further studies. We suggest that the full version of TROG-2 should be used for a comprehensive assessment of sentence comprehension with views to devise tailor-made intervention programs, monitor the progression of language deterioration, and apply cross-linguistic and basic research. For these purposes, TROG-2 or TROG2-Br should be applied solely in a session or split into two sessions in order not to cause fatigue and overload attentional resources.

This study has some limitations. Although the PPA sample is large compared to previous studies, our samples of PPA-NF/A and PPA-L are small, and it is a clinic-based cohort. The control group was recruited for convenience whereas a population-based study would be more appropriate to generate norms for the use of the test. The full validation of TROG2-Br is still in progress, and it is necessary to establish education adjusted cut-off scores as well as interrater and test-retest reliability. The PPA sample was assessed over the years, and brief and full neuropsychological assessments were not comparable or available to correlate TROG2-Br with performance in other cognitive domains. Future studies need to address the relationship between performance in TROG2-Br and measures of attention, working memory, and executive functions to better comprehend the nature of the deficits in PPA individuals, who are speakers of Portuguese, and the impacts of education on the performance in the task. Despite these caveats, our findings with TROG2-Br have shown that this tool may be helpful to detect and characterize sentence comprehension difficulties in PPA and no similar tool is available for this type of assessment in Brazilian Portuguese. Some studies have used TROG to characterize and compare phenotypes in neurodegenerative frontotemporal syndromes as a source for predicting neuropathology and progression of disease (24, 41, 58–60, 117). The availability of TROG2-Br may facilitate similar studies in the speakers of Portuguese that may contribute to the understanding of the relationship between language, perception, and motor functions.

Grammar and syntax require specifically designed tasks for assessment (10, 13, 15). Most studies in PPA have focused on grammar production but sentence comprehension is affected due to different underlying reasons in all PPAs. Quantification and characterization of sentence comprehension may help to develop more efficient tailor-made programs to benefit communication in these patients (10, 118). Future studies in Latin America should address other measures of sentence production and sentence comprehension in Portuguese to improve the care and quality of life of individuals with PPA as well as to benefit cross-language and cross-cultural clinical research on language comprehension.

## Data Availability Statement

The raw data supporting the conclusions of this article is available in the supplementary material, further inquiries can be directed to the corresponding author.

## Ethics Statement

The study was approved by the Ethics Committee of the University of the City of São Paulo (CAAE 0110.0.186.000-11/Research Protocol 13622453) and by the Ethics Committee of the School of Medicine of Federal University of Minas Gerais (CAAE 60390116.9.0000.5149/ Research Protocol 2.018.855). The patients/participants provided their written informed consent to participate in this study.

## Author Contributions

MTCG, YZ, THM, and PC contributed to the conception of the study. MTCG, IJA, DS, and THM wrote the manuscript that was revised by all the co-authors. THM, AC, IJA, MTCG, and PC assessed and analyzed TROG2-Br of PPA individuals. RO, MTCG, DS, and YZ assessed and analyzed the data from healthy elderly controls. All authors contributed to the analysis of data reported in the manuscript.

## Funding

The study received funding from CAPES Grant Number BEX 4335/074 and 001. FAPESP: Grant Number 11/08779-1. RO and DS received funding from UFABC for their MSc and PhD programs, respectively.

## Conflict of Interest

The authors declare that the research was conducted in the absence of any commercial or financial relationships that could be construed as a potential conflict of interest.

## Publisher's Note

All claims expressed in this article are solely those of the authors and do not necessarily represent those of their affiliated organizations, or those of the publisher, the editors and the reviewers. Any product that may be evaluated in this article, or claim that may be made by its manufacturer, is not guaranteed or endorsed by the publisher.
