# Supplementary material for: Sentence Comprehension in Primary Progressive Aphasia: A Study of the Application of the Brazilian Version of the Test for the Reception of Grammar (TROG2-Br)
Source: Front Neurol. 2022 May 16;13:815227. doi: 10.3389/fneur.2022.815227 (PMC9149594; doi:10.3389/fneur.2022.815227)
Supplement: Supplementary file 1 [file Data_Sheet_1.docx]

**Supplementary Material**

**Table S1: Individual performance of controls on cognitive screening and TROG2-Br**

| ID | Sex | Education | Age | MMSE | ACE-R | Attention | Fluency | Language | Memory | Visuospatial | TROG2-Br (i) | TROG2-Br (b) |
| --- | --- | --- | --- | --- | --- | --- | --- | --- | --- | --- | --- | --- |
| 1 | male | 2 | 70 | 25 | 68 | 15 | 9 | 15 | 16 | 13 | 72 | 13 |
| 2 | male | 2 | 78 | 24 | 68 | 15 | 12 | 14 | 16 | 11 | 72 | 14 |
| 3 | female | 2 | 92 | 26 | 71 | 16 | 10 | 18 | 17 | 10 | 76 | 16 |
| 4 | male | 3 | 64 | 26 | 86 | 15 | 10 | 26 | 19 | 16 | 57 | 6** |
| 5 | female | 3 | 69 | 30 | 81 | 18 | 8 | 21 | 22 | 12 | 58 | 5** |
| 6 | female | 3 | 75 | 23 | 69 | 15 | 11 | 15 | 17 | 11 | 63 | 10** |
| 7 | female | 3 | 70 | 29 | 80 | 18 | 10 | 23 | 13 | 16 | 63 | 11 |
| 8 | female | 3 | 79 | 25 | 72 | 15 | 8 | 18 | 20 | 11 | 67 | 9** |
| 9 | female | 3 | 66 | 28 | 90 | 17 | 8 | 26 | 25 | 14 | 67 | 12 |
| 10 | female | 3 | 80 | 24 | 71 | 15 | 8 | 19 | 17 | 12 | 70 | 14 |
| 11 | female | 3 | 80 | 27 | 75 | 16 | 10 | 21 | 20 | 8 | 72 | 13 |
| 12 | female | 3 | 80 | 24 | 69 | 14 | 10 | 18 | 19 | 8 | 72 | 13 |
| 13 | male | 3 | 71 | 25 | 68 | 17 | 8 | 14 | 18 | 11 | 72 | 13 |
| 14 | female | 3 | 84 | 27 | 70 | 15 | 10 | 19 | 16 | 10 | 72 | 14 |
| 15 | female | 3 | 71 | 25 | 73 | 17 | 9 | 17 | 19 | 11 | 73 | 14 |
| 16 | female | 3 | 63 | 24 | 76 | 14 | 9 | 23 | 16 | 14 | 74 | 15 |
| 17 | female | 3 | 72 | 24 | 78 | 15 | 8 | 19 | 22 | 14 | 75 | 15 |
| 18 | female | 3 | 71 | 25 | 79 | 15 | 10 | 22 | 18 | 14 | 78 | 19 |
| 19 | female | 4 | 62 | 26 | 67 | 15 | 5 | 18 | 15 | 14 | 51 | 4** |
| 20 | male | 4 | 72 | 28 | 76 | 17 | 9 | 22 | 15 | 13 | 72 | 12 |
| 21 | male | 4 | 79 | 24 | 80 | 15 | 10 | 22 | 18 | 15 | 72 | 14 |
| 22 | male | 4 | 90 | 27 | 77 | 17 | 4 | 23 | 19 | 14 | 73 | 14 |
| 23 | female | 4 | 62 | 28 | 91 | 17 | 11 | 26 | 24 | 13 | 76 | 16 |
| 24 | male | 4 | 74 | 28 | 83 | 17 | 6 | 24 | 22 | 14 | 76 | 16 |
| 25 | female | 4 | 79 | 29 | 91 | 18 | 12 | 25 | 21 | 15 | 76 | 17 |
| 26 | male | 4 | 75 | 27 | 84 | 17 | 8 | 24 | 22 | 13 | 76 | 17 |
| 27 | female | 4 | 68 | 30 | 85 | 13 | 8 | 25 | 23 | 16 | 77 | 17 |
| 28 | male | 4 | 72 | 30 | 77 | 18 | 6 | 19 | 22 | 12 | 77 | 17 |
| 29 | female | 4 | 74 | 28 | 87 | 17 | 8 | 26 | 23 | 13 | 77 | 17 |
| 30 | female | 4 | 71 | 30 | 94 | 18 | 10 | 26 | 25 | 15 | 77 | 18 |
| 31 | female | 4 | 69 | 29 | 94 | 18 | 12 | 25 | 25 | 14 | 78 | 18 |
| 32 | female | 4 | 78 | 27 | 84 | 18 | 9 | 25 | 16 | 16 | 78 | 18 |
| 33 | male | 4 | 74 | 27 | 87 | 17 | 9 | 26 | 21 | 14 | 78 | 19 |
| 34 | female | 4 | 80 | 29 | 91 | 18 | 9 | 25 | 23 | 16 | 80 | 20 |
| 35 | female | 4 | 77 | 29 | 87 | 18 | 13 | 25 | 20 | 11 | 80 | 20 |
| 36 | female | 4 | 70 | 25 | 88 | 16 | 10 | 26 | 21 | 15 | 80 | 20 |
| 37 | female | 5 | 72 | 24 | 77 | 16 | 11 | 21 | 17 | 12 | 72 | 13 |
| 38 | female | 6 | 63 | 25 | 84 | 15 | 11 | 26 | 16 | 16 | 54 | 5** |
| 39 | female | 6 | 60 | 28 | 94 | 16 | 10 | 26 | 26 | 16 | 73 | 13 |
| 40 | female | 6 | 77 | 29 | 93 | 18 | 10 | 26 | 25 | 14 | 74 | 14 |
| 41 | female | 6 | 70 | 28 | 94 | 16 | 13 | 26 | 26 | 13 | 76 | 16 |
| 42 | male | 6 | 77 | 28 | 91 | 17 | 12 | 25 | 25 | 12 | 76 | 17 |
| 43 | male | 6 | 80 | 28 | 75* | 17 | 7 | 22 | 17 | 12 | 76 | 17 |
| 44 | female | 7 | 62 | 26 | 85 | 17 | 9 | 24 | 21 | 14 | 65 | 8** |
| 45 | male | 7 | 75 | 27 | 86 | 16 | 10 | 22 | 25 | 13 | 67 | 10** |
| 46 | female | 7 | 66 | 29 | 90 | 17 | 11 | 23 | 24 | 15 | 77 | 17 |
| 47 | male | 8 | 67 | 28 | 90 | 18 | 6 | 25 | 25 | 16 | 75 | 15 |
| 48 | male | 8 | 85 | 28 | 90 | 17 | 12 | 26 | 19 | 16 | 76 | 16 |
| 49 | female | 8 | 60 | 27 | 74* | 15 | 9 | 23 | 15 | 12 | 77 | 17 |
| 50 | female | 8 | 61 | 29 | 95 | 18 | 10 | 26 | 25 | 16 | 80 | 20 |
| 51 | male | 11 | 69 | 30 | 83* | 16 | 14 | 26 | 12 | 15 | 71 | 13 |
| 52 | male | 11 | 60 | 27 | 81* | 17 | 9 | 23 | 21 | 10 | 74 | 14 |
| 53 | female | 11 | 84 | 28 | 89 | 18 | 9 | 25 | 21 | 16 | 78 | 18 |
| 54 | female | 11 | 61 | 29 | 93 | 18 | 11 | 25 | 25 | 14 | 79 | 19 |
| 55 | female | 15 | 80 | 27 | 90 | 17 | 13 | 25 | 22 | 13 | 68 | 11 |
| 56 | female | 15 | 74 | 28 | 90 | 16 | 14 | 26 | 21 | 13 | 70 | 12 |
| 57 | female | 15 | 63 | 30 | 95 | 18 | 13 | 26 | 25 | 13 | 71 | 13 |
| 58 | female | 15 | 79 | 30 | 94 | 18 | 11 | 25 | 25 | 15 | 73 | 14 |
| 59 | female | 15 | 67 | 29 | 94 | 17 | 13 | 26 | 26 | 12 | 73 | 14 |
| 60 | female | 15 | 68 | 29 | 94 | 18 | 10 | 26 | 24 | 16 | 73 | 15 |
| 61 | male | 15 | 79 | 27 | 82* | 18 | 9 | 25 | 17 | 13 | 74 | 15 |
| 62 | male | 15 | 69 | 29 | 90 | 18 | 13 | 26 | 20 | 13 | 74 | 15 |
| 63 | female | 15 | 70 | 27 | 93 | 16 | 11 | 26 | 24 | 16 | 78 | 18 |
| 64 | female | 15 | 91 | 30 | 97 | 18 | 11 | 26 | 26 | 16 | 78 | 18 |
| 65 | female | 15 | 64 | 29 | 96 | 18 | 11 | 26 | 25 | 16 | 78 | 18 |
| 66 | female | 15 | 84 | 28 | 93 | 17 | 13 | 26 | 23 | 14 | 79 | 19 |
| 67 | female | 15 | 64 | 27 | 86 | 15 | 8 | 25 | 22 | 16 | 79 | 19 |
| 68 | female | 15 | 61 | 30 | 96 | 18 | 13 | 25 | 25 | 15 | 79 | 19 |
| 69 | female | 15 | 78 | 28 | 95 | 18 | 12 | 26 | 23 | 16 | 80 | 20 |
| 70 | female | 15 | 63 | 30 | 95 | 18 | 12 | 26 | 25 | 14 | 80 | 20 |
| 71 | female | 15 | 77 | 29 | 89 | 17 | 9 | 26 | 25 | 12 | 80 | 20 |
| 72 | female | 17 | 78 | 29 | 93 | 18 | 12 | 26 | 25 | 12 | 75 | 15 |
| 73 | female | 17 | 72 | 30 | 92 | 18 | 11 | 26 | 21 | 16 | 76 | 16 |
| 74 | female | 17 | 75 | 29 | 94 | 18 | 11 | 25 | 24 | 16 | 80 | 20 |

ID: identification; ACE-R: Addenbrooke’s Cognitive Examination Revised (ACE-R); MMSE: Mini-Mental State Examination;(i): number of items correctly responded in TROG2-Br; (b): number of blocks correctly responded in TROG2-Br.

* Score compatible to cognitive-impairment- no dementia, education-adjusted cut-off scores

**Extremely low performance on TROG2-Br (≤ 50% of the test)

**Table S2: Individual performance of individuals with PPA in TROG2-Br**

| Cases | PPA | A | B | C | D | E | F | G | H | I | J | K | L | M | N | O | P | Q | R | S | T | Total | Total |
| --- | --- | --- | --- | --- | --- | --- | --- | --- | --- | --- | --- | --- | --- | --- | --- | --- | --- | --- | --- | --- | --- | --- | --- |
|  |  | N/P | N/P | N/P | N/P | N/P | N/P | N/P | N/P | N/P | N/P | N/P | N/P | N/P | N/P | N/P | N/P | N/P | N/P | N/P | N/P | Items | Blocks |
|  |  |  |  |  |  |  |  |  |  |  |  |  |  |  |  |  |  |  |  |  |  |  |  |
| 1 | L | P | P | 2 | P | 2 | 1 | 3 | 4 | 2 | 3 | 2 | 3 | 2 | 3 | 1 | 1 | 3 | 2 | 4 | 3 | 39 | 3 |
| 2 | L | 1 | 1 | 1 | P | 1 | 4 | 2 | 3 | 1 | 3 | 2 | 3 | 2 | 3 | 2 | 1 | 2 | 1 | 3 | 2 | 42 | 1 |
| 3 | L | P | P | P | P | P | P | P | 1 | P | P | 2 | 2 | 1 | 2 | 1 | P | 2 | P | 1 | 4 | 64 | 11 |
| 4 | L | P | P | P | P | P | P | P | P | P | P | 2 | P | P | 2 | P | 1 | 2 | 2 | 1 | 2 | 68 | 13 |
| 5 | L | 1 | 2 | 3 | P | P | 1 | 3 | P | 2 | P | 3 | 4 | 1 | 1 | P | 1 | 2 | 1 | 3 | 4 | 48 | 5 |
| 6 | Mx | 1 | 2 | 3 | 2 | 2 | 2 | 4 | 3 | 3 | 3 | 2 | 3 | 2 | 3 | 3 | 3 | 3 | 3 | 3 | 4 | 26 | 0 |
| 7 | Mx | 1 | 1 | 1 | 1 | 1 | 1 | 3 | 1 | P | 4 | 4 | 4 | 4 | 4 | 4 | 4 | 4 | 4 | 4 | 4 | 26 | 1 |
| 8 | Mx | 1 | 1 | 1 | P | 3 | 3 | 4 | 2 | 3 | 3 | 4 | 2 | 2 | 4 | 3 | 4 | 3 | 1 | 3 | 2 | 31 | 1 |
| 9 | Mx | P | 3 | 2 | P | 2 | 3 | 3 | 1 | 1 | 3 | 2 | 4 | 2 | 3 | 3 | 2 | 3 | 3 | 4 | 4 | 32 | 2 |
| 10 | Mx | P | 1 | 1 | P | 1 | P | 3 | P | 1 | 3 | 2 | 4 | 4 | 2 | 4 | 3 | 3 | 3 | 2 | 4 | 39 | 4 |
| 11 | Mx | P | 2 | 2 | 1 | 1 | 3 | 2 | 1 | 2 | 1 | 4 | 3 | 3 | 1 | 2 | 3 | 2 | 2 | 3 | 1 | 41 | 1 |
| 12 | Mx | P | P | 2 | P | 1 | P | 2 | P | 1 | 4 | 3 | 2 | 1 | 2 | 2 | 3 | 2 | 2 | 4 | 3 | 46 | 5 |
| 13 | Mx | P | P | 1 | P | 1 | 1 | 1 | 3 | P | P | 2 | 3 | 2 | P | P | 1 | 2 | 2 | 2 | 3 | 56 | 7 |
| 14 | Mx | P | P | P | P | P | P | P | P | P | P | 3 | 2 | P | 1 | P | P | 1 | 1 | 2 | 3 | 67 | 13 |
| 15 | Mx | P | P | P | P | P | P | P | P | 1 | P | P | 2 | P | 1 | P | P | 1 | 1 | P | 3 | 71 | 14 |
| 16 | Mx | P | P | P | P | P | P | P | P | P | P | P | P | P | 1 | P | P | P | P | P | P | 79 | 19 |
| 17 | NF/A | 1 | 4 | 3 | 2 | 2 | 3 | 2 | 2 | 2 | 4 | 1 | 3 | 2 | P | 4 | 4 | 2 | 2 | 3 | 4 | 30 | 1 |
| 18 | NF/A | P | P | 1 | 2 | 1 | 2 | 2 | 2 | 3 | 2 | 3 | 2 | 3 | 3 | 3 | 3 | 1 | 1 | 3 | 4 | 39 | 2 |
| 19 | NF/A | P | P | 1 | P | 1 | P | 2 | P | 2 | 1 | 3 | 4 | 2 | 2 | P | 2 | 2 | 3 | 4 | 4 | 47 | 6 |
| 20 | NF/A | P | P | 2 | P | 2 | P | 1 | P | 1 | 1 | 2 | 2 | 3 | 2 | 1 | 2 | P | 2 | 3 | 3 | 53 | 6 |
| 21 | NF/A | P | P | P | P | 1 | P | P | 3 | 1 | P | 3 | 2 | 1 | 2 | P | 1 | 3 | 2 | 3 | 3 | 55 | 8 |
| 22 | NF/A | P | P | P | P | P | P | P | P | P | P | 1 | P | 1 | P | P | P | P | P | P | 2 | 76 | 17 |
| 23 | S | 1 | 2 | 1 | P | 1 | 2 | 2 | 1 | 2 | 3 | 3 | 4 | 2 | 2 | 3 | 3 | 4 | 1 | 3 | 4 | 36 | 1 |
| 24 | S | P | 1 | 1 | 2 | 1 | 2 | 2 | 4 | 3 | 2 | 3 | 1 | 2 | 3 | 2 | 3 | 1 | 2 | 2 | 3 | 40 | 1 |
| 25 | S | P | P | 1 | P | 3 | 1 | 2 | 1 | 2 | 4 | 2 | 2 | 2 | 2 | 3 | 2 | 2 | 2 | 3 | 3 | 43 | 3 |
| 26 | S | P | P | 1 | P | P | 1 | 2 | 1 | 2 | 3 | 2 | 3 | 4 | P | 2 | 1 | 4 | 2 | 3 | 4 | 45 | 5 |
| 27 | S | P | P | 1 | P | P | P | 1 | P | 1 | P | 2 | 2 | P | P | P | 1 | 1 | 3 | 3 | 3 | 62 | 9 |
| 28 | S | P | P | 1 | 1 | P | 2 | P | P | P | 2 | 4 | 1 | P | P | 1 | P | 1 | P | 3 | 2 | 62 | 10 |
| 29 | S | P | P | 1 | P | P | P | P | 1 | 1 | P | 2 | 2 | P | P | P | P | 1 | P | 2 | 2 | 68 | 12 |
| 30 | S | 1 | P | P | P | P | P | P | 3 | P | P | 3 | P | P | P | P | P | P | P | 2 | 3 | 68 | 14 |
| 31 | S | P | P | P | P | P | P | P | 1 | P | P | P | 1 | P | 1 | P | P | 1 | P | 1 | 2 | 73 | 14 |
| 32 | S | 1 | P | 1 | P | P | P | P | P | P | P | 1 | 1 | P | P | P | P | 1 | P | P | 2 | 73 | 14 |
| 33 | S | P | P | 1 | P | P | 1 | P | P | P | P | P | P | 1 | P | P | 1 | P | P | P | 1 | 75 | 15 |
| 34 | S | P | P | P | P | P | P | P | P | P | P | 1 | P | P | P | P | P | P | P | P | P | 79 | 19 |

L: Logopenic variant of Primary Progressive Aphasia; S: Semantic variant of Primary Progressive Aphasia; NF/A: Nonfluent/Agrammatic variant of Primary Progressive Aphasia; Mx: Mixed (unclassified) variant of Primary Progressive Aphasia. N/P: number of errors in the block/passed block (all correct). P: passed block

**Figure S1: Individual performance of controls and individuals with PPA in TROG2-Br – passed blocks**

Insert FigureS1

PPA: Primary Progressive Aphasia group; PPA-L: Logopenic variant of Primary Progressive Aphasia; PPA-S: Semantic variant of Primary Progressive Aphasia; PPA-NF/A: Nonfluent/Agrammatic variant of Primary Progressive Aphasia; PPA-Mx: Mixed (unclassified) variant of Primary Progressive Aphasia. SD: Standard Deviation. *Mann-Whitney U and p = comparisons between PPA group and Control group. 8 controls did not present errors, so for error analysis the number of controls is 66.
